# Time, scarcity, and abundance

**DOI:** 10.3389/frma.2022.974706

**Published:** 2022-08-31

**Authors:** Shubha Ghosh

**Affiliations:** Syracuse University College of Law, Syracuse, NY, United States

**Keywords:** regulation, social media, copyright, intellectual property, economics of growth and technology, economic theory

## Abstract

Scarcity abounds in law just as abundance is subject to law's limitations. This Article builds on legal theory, economics, and social psychology to present the dialectic of scarcity and abundance as they interplay in our relationship to information and time. This Article has made two overarching arguments: one about scarcity, abundance, and regulation generally and a second about time as an instrument of regulation subject to terms of scarcity and of abundance. The first argument is that scarcity and abundance are rhetorical constructs that inform different regulatory institutions. Scarcity traditionally has mapped onto limits on freedom. Abundance, by contrast, props freedom's unlimited potential. Under the language of scarcity, limits promote outcomes, for example through rights to exclude, deprivation of a benefit, or imposition of a burden. Under the language of abundance, identified freedoms promote outcomes through rights of access or rights to use. Scarcity is distinct from absolute deprivation, and abundance, from unbounded and infinite possibility. Each are building blocks understood relative to the goals of institutional design. Furthermore, scarcity and abundance have an intertwined relationship, a dialectic of famine and plenty. Similarly, freedom and limitations coexist each supporting the other. The second argument of this Article is that time as an instrument of regulation illustrates the uses of scarcity and abundance. Time can be regimented to regulate activities such as work, travel, diet, reproductive rights, social relations, and interaction with media. Time can also be liberating, seemingly abundant using perpetuities, technologies for fast forwarding, rewinding, or shifting content, and increases in the velocity of access and movement. Information retrieval, processing, and sharing are connected to time. It is no surprise that reform proposals for the problems confronting the information economy rest up regulation of time. This Article has demonstrated what these reform proposals share is an attempt to make time scarce, to return to perhaps an idealized era of regimented broadcast within an era of multivalent technological means for information creation and dissemination. But imposing scarcity on abundance ignores the deeper challenges of information glut and distortion: how to manage and assess content.

## The allure of scarcity and abundance in intellectual property law

Scarcity abounds in law just as abundance is subject to law's limitations. This Article builds on legal theory, economics, and social psychology to present the dialectic of scarcity and abundance as they interplay in our relationship to information and time.

To examine the concept of scarcity is to challenge the foundation of law. After all, law is often about limitations: the boundaries of a prison, the meting out of a sentence. Intellectual property law, for example, is most notably about limits, whether with respect to the limited times of the exclusive rights of patents and copyrights, the survival of rights of publicity beyond the death of the public figure, or the abandonment of trademarks and trade secrets. The right to exclude under any of the five prominent intellectual property regimes places limits on consumers, makers, and creators, requiring each to negotiate with the owner or the search for alternatives to work around the limitations. One cannot meaningfully engage with the law without confronting the concept of scarcity.

But law is not only about limitations. Law also enables freedom, whether the freedom to travel, the freedom to speak, the freedom to exchange, or the freedom to invent. With this freedom comes a form of abundance. Legal rights proliferate: the right to be free from censorship of one's book or movie becomes the right to spend on campaign finance. There is nothing inevitable about how rights reproduce and multiply. But there is a noticeable inexorability to rights proliferating. A tangible manifestation is the exponentially growing size of federal and state codes that spell out rights and duties. To take another example: new technologies broaden the scope of rights, such as freedom of association and communication and the freedom from unwarranted searches and seizures. Developments of these new technologies spark new intellectual property rights and duties.

This Article explores the dialectic of scarcity and abundance in law, especially in the law surrounding information and communication technologies. As the previous two paragraphs set forth, scarcity and abundance in law is about law's conflicting roles in limiting and expanding rights. More concretely, this dual role translates into how we gauge the consequences of rights and remedies for the design of legal and social institutions. We talk about these consequences in debates over legal policy. Often the rhetoric of these debates speaks to legal rights being too strong or too weak, too broad or too narrow. Such language, however, can be confusing and unhelpful. What is the scale for determining the strength and weakness of rights? What is the perspective for saying a set of rights is too broad or narrow? Underlying the rhetoric about the size of rights is a difficulty in confronting the concepts of scarcity and abundance. To confront these concepts is to enliven ongoing policy debates in law.

How does the dialectic of scarcity and abundance play out in legal policy debates? I confront this question as follows. The first step is a return to the foundation of scarcity in the discipline of economics. The second step is to show how this foundational concept has developed in policy debates over abundance and technological change. Against this background on the concepts of scarcity and abundance, I connect this background to debates within law, specifically copyright, information, and communication technologies. A critical insight is that scarcity and abundance in the space of information relate to questions of time allocation and management. Time, as I show, is an instrument for regulation in the information economy. The final step is to connect the play of scarcity and abundance in law to current debates over the regulation of social media through what I call time architecture, pointing toward a need to think beyond scarcity and abundance to focus on political and policy questions of institutional design.

## Lionel Robbins, scarcity, and the focus on means

Our conception of scarcity has its roots in the 1932 definition of economics, offered by Lionel Robbins: “Economics is the science which studies human behavior as a relationship between ends and *scarce* means which have alternative uses” (Robbins, 1932). As a marker for a disciplinary boundary, the word scarce, here an adjective, sets out one of the objects of study of economics, means or tools to reach certain ends or goals. While the tools are scarce, the ends or uses of the tools may or may not be scarce or limited. This focus on instrumentality identifies what is distinctive about the study of economics; it is the study of how to reach a result when there are few options. Within law, an analogous problem is how to use legal institutions and processes to reach certain ends, whether dispute resolution, the fulfillment of transactions, or the more elusive goal of justice. It is not surprising that economics has had an influence on law, and one might notice that economics and law both have roots in what was once called moral philosophy (Smith, 1762; Robbins, 1932, p. 16; Hausman and McPherson, 2016; Malloy, 2021).

Scarce as a modifier of the word means leads to scarcity, a more abstract concept. Modern economics takes scarcity as a given phenomenon with which economists must reckon. We can find scarcity in many circumstances. A household has a limited annual income with which to satisfy the wants of its members. An organization, such as a university or a manufacturing concern, must sort out how to use its people and tangible assets in order to reach its goals, whether the education of smart students or the making of smart phones. A nation must manage its natural resources, be it minerals, oil, forests, or water, to meet the consumption needs of its citizens and as possible means for producing wealth. What Robbins sought to do with his definition is to broaden the scope of economics as a discipline beyond a narrow focus on identifying the causes of material wealth (Robbins, 1932, p. 16). His broadening of economic methodology was a response to several traditions, first, the mercantilist tradition with its emphasis on the capture of wealth through trade and conquest; second, the tradition initiated by Adam Smith that looked to human industry as the source of the wealth of nations; and third, the marginalist revolution initiated by Alfred Marshall in the late nineteenth century that grounded economics in methods of optimization. Robbins was identifying economics as a discipline attuned to broader questions of decision making in many contexts beyond the accumulation of material wealth and individual choice. Economics as confronting the problem of connecting scarce means to broad ends was a systematic discipline with applications beyond what the mercantilists, Adam Smith, or Alfred Marshall imagined.

Robbins' identification of scarce means and broad ends carries over to other disciplines where economics has application. His definition sees economics as a method for planning and consciously a response to those who at the time were advocating strong centralized planning. Robbins' definition provides a foundation for management science beyond that of centralization. As a management science, economics informs the directors of companies, planners on local zoning boards, heads of government agencies (national and regional), and smart household shoppers. The discipline can also guide attorneys as they advise clients through litigation or through complex transactions. Law and Economics in its original formulation identified how judges did and should use economic thinking to command the tools of law (rights and remedies) to reach the ends of specific areas of law (compensation for injury, the fulfillment of promises). Throughout all these extensions from Robbins' brief definition we see the ubiquity of scarcity as a concept.

Robbins builds from his definition of economics a logical and deductive system for studying exchange in the economy. He does not, however, talk about law, except in one surprising instance that has relevance for this Article. His example is copyright. The example begins with a seemingly bizarre point in response to those who reduced the field of economics purely to the study of material wealth. Robbins, citing Professor Edwin Cannan, his mentor, quotes “'Did Bacon write Shakespeare?' was not an economic question…[but] the controversy would have an economic side if copyright were perpetual, and the descendants of Bacon and Shakespeare were disputing the ownership of the plays” (Robbins, 1932, p. 16, citing Cannan, 1928). Robbins asks why does this question become a matter of economics if it were a dispute over extant rights. His response is that,

the question [of authorship] has an economic aspect simply and solely because
the copyright laws supposed would make the use of the plays scarce in relation
to the demand for their use, and would in turn provide their owners with command
over scarce means of gratification which otherwise would be differently distributed (Robbins, 1932, p. 16, citing Cannan, 1928).

What makes authorship a question of economics, according to Robbins, is the scarcity that copyright creates. Through this legally created scarcity, the copyright owner decides what uses of the play can be made in order to satisfy demand. Copyright, in other words, illustrates the economic problem of managing scarce uses to satisfy the gratification from watching a Bacon (or Shakespeare) owned play. The cultural question is a separate question^1^ from the questions of economics, which is about who directs the use of a scarce means for entertainment and how these ends are met.

Scholars of intellectual property will not find much new in Robbins' analysis although it is surprising to see the example come up in a book directed at economists. Law as a source of artificial scarcity is well-recognized as is the role of the copyright owner in determining how the uses of the copyrighted work might be directed. Although there are no details here about licensing, fair use, exhaustion or other doctrinal details, the image of the copyright owner mediating access to the work with users' needs is familiar. Robbins mention of “otherwise would be differently distributed” speaks to alternate means for satisfying audiences beyond copyright, perhaps through a patronage system or the public domain. Robbins was not concerned with those details except to suggest that these alternative arrangements would have different distributional effects, by which he means who enjoys the benefits and bears the costs. Contemporary scholars would point out that these alternatives to copyright have more than distributional impacts; they would affect the quality and volume of the work^2^. But Robbins' point survives as an economics matter, copyright is about managing scarce uses to satisfy demand.

However, there are many dimensions to copyright that belie scarcity. Robbins spoke of demand for the plays implicitly as forms of private consumption. We might think of these plays as public goods, in many senses of the term. They are consumed publicly or jointly. They are also part of cultural heritage that is generally available through the public domain. For Robbins, these nuances would be irrelevant to the economic question. Whether described as private or public, these are the ends. Economics' focus is on scarce means, which in the case of copyright are the exclusive rights that must be authorized by the owner. This distinction between means and ends is a limitation on the economic methodology. Perhaps ends themselves are scarce, in the sense that society needs a mechanism to choose what ends to pursue. Should we pursue entertainment or should we pursue science? Should we build a theater or should we build a ballpark? These questions point to the larger concern that there are limits to what goals we can pursue and attain. Furthermore, the economic view is constraining by assuming that means are limited. Copyright is not the only option. It can be combined with other institutions. Or perhaps replaced altogether. Too narrow a focus on scarce means and ends through instrumental thinking may be the problem and not the solution. As we shall see in the next section, these limitations from the concept of scarcity are what makes arguments based on abundance more appealing.

Robbins, however, was aware of the problems with his conception of economic methodology and its applications. As he admits, “it is clearly necessary to assume a social order within which the valuations based upon it may show themselves in tendencies to action” (Robbins, 1932, p. 93). In other words, means-end rationality is contextual. Robbins use of the word “assume” is unfortunate. Background context of social order is very much real, even though it is also the product of human decision-making. Robbins goes on to say, in elaborating on the economic theory of exchange:

In the theory of simple exchange, for instance, we assume that Primus is free
to acquire corn from Secundus by offering him wine. But we do not necessarily
assume that he is free to acquire corn by killing him or otherwise doing him violence.
We assume a legal framework of economic activity. This framework, as it were,
limits the area within which the valuations of the economic subjects may influence
their action. It prescribes a region in which one is *not* free to adopt all possible
expedients; and these prescriptions are assumed in the discussion of what happens
in the residual area of free action (Robbins, 1932, p. 93–94).

As we saw in his discussion of copyright, law creates its own limitations within which economic exchange operates as pursuit of ends through the scarce means of producing, buying, and selling. Robbins, however, does not discuss how to assess these limitations explicitly. But presumably the economic methodology he proposes offers an instrumental way to formulate these background laws. It should be pointed out that throughout his career, Robbins was active in policy debates during and after World War Two in the United Kingdom.

In providing a foundation for the concept of scarcity, Robbins' emphasis is on limitations, what decisions must be foregone, what choices must be made. He was aware of how technological change can remove these limitations. But he was skeptical of the idea that new technologies require new economics. For Robbins, the fundamental problem of instrumental reasoning and scarcity was persistent.

It is perfectly true that with the advance of modern technologies, the provision of the
most elementary requirements of “material welfare” has come to demand a diminishing
proportion of the powers of production at the disposal of the human race. But it is not in
the least true the phenomenon of prices and costs, incomes and capitalizing rates, which
are the central preoccupation of the Economics of an exchange economy, have shown
any tendency to disappear or to lose their practical significance (Robbins, 1932, p. 97–98).

Robbins is claiming that the question of scarcity is salient to understand the advances of technologies even as technological advances may provide abundance. Scarcity is persistent; there are always constraints to decision making even if there is seeming abundance. Robbins' analysis sets up a dialectic between scarcity and abundance that transforms how we identify new means to satisfy broad, perhaps even growing, ends. To appreciate this dialectic, and its relevance to current debates over information, communication technologies, and copyright, we need to assess our understanding of abundance, the subject of the next section.

## Ester Boserup (and others), abundance, and the problem of ends

In 1980, economist Julian Simon accepted a bet from biologist Paul Ehrlich that put the concept of scarcity to the test. At the heart of the bet was a prediction about changes in the price of a bundle of commodities, nickel, copper, chromium, tin, and tungsten, at the end of a ten-year period. Ehrlich, the author of The Population Bomb which revived the Malthusian proposition that population growth will overburden natural resources, bet on the side of scarcity, predicting that the price will rise after ten years due to shortages arising from demand outstripping supply. Simon took the side of abundance, betting that human ingenuity in managing scarcity through technology would lead to a fall in price. At the end of the decade, Simon had famously won the bet (Worstatt, 2012).

Several explanations are offered for why Simon won. His success was partly a matter of lucky timing. Prices of the chosen commodities rose in the 1990's and 2000's, suggesting that Ehrlich would have won if the two had bet over a fifteen- or twenty-year period. Several factors made the 1990 an unusual year. A tin cartel had gone bankrupt, correcting artificially monopolized prices. The Soviet Union, a producer of the non-ferrous metals in the bundle, collapsed causing the world market to be flooded with these metals as domestic demand for them dropped. The market, however, corrected in the 1990's favoring Ehrlich's side of the bet (Worstatt, 2012). Perhaps the argument for scarcity is stronger than Simon's win would suggest.

But favoring Simon's claims about the power of human ingenuity was the development in the 1980's of solvent extraction and electo-winning, innovative processes for extracting copper from copper oxide, copiously stored in mountains but difficult to mine without the technological advance (Worstatt, 2012). This technological advance supports what we can call the argument for abundance, as a counter to scarcity. As a proponent of abundance, Simon is sometimes described as a cornucopian, a believer in the unlimited possibility of technology to satisfy human needs and wants. Contrary to Robbins' emphasis on scarce means, a cornucopian would emphasize that means are not scarce. To quote the cliché, necessity fosters invention, especially where the necessity may result from scarcity.

Although the cornucopian vision of unlimited possibility can readily turn utopian, developments in science and its technological fruits bolster such optimism. Post-World War Two, agriculture was in crisis, causing prognosticators to predict global famine, concentrated in developing countries. Dr. Norman Borlaug devised new methods for farming, creating high-breed plants with greater nutritional value. The Green Revolution abated fears of world-wide starvation. In the 1970's and 1980's, developments in genetic technology further improved the quality of rice and other grains, allowing many developing countries to become self-sufficient and some, even exporters, of agricultural products. However, these developments fueled new concerns as farmers were displaced by these technological developments. While government subsidies aided displaced farmers, fears of a new crisis arose as farms increased in scale and size reducing the income of smaller plots owned by independent farmers. One response to these concerns was reforming the agricultural sector in some countries to allow family farms to transition to more profitable enterprises. Another response was technological, specifically finding ways to enhance the natural process of photosynthesis to make plants more efficient in how they process carbon dioxide through improvements in the design of leaves and the underlying biochemistry (Kolbert, 2021). Corcnucopians may mythologize the power of technology, but its influence, however gradual and unpredictable, cannot be denied.

A parallel project to this Article discusses how crisis can fuel invention and innovation. This concurrent work examines the current COVID pandemic (and antecedents in the polio and AIDS crises) and its challenge for invention, innovation, and government policy responses to patent rights and drug approval. Crises illustrate the interplay between scarcity and abundance, the subject of this Article. What the two projects share, in part, is a critical reliance on the work of Ester Boserup, a researcher who in many ways challenged the perspectives of doomsayers like Ehrlich and utopians like Simon.

Ester Boserup, often identified as an agricultural economist and scholar of economic development, provides a theoretical framework, that is empirically based, for specifying how crisis, invention, and innovation mix. Dr. Boserup's insight is the idea of induced innovation. Her idea was a response to the Mathusian trap that arose from human population growing geometrically while agricultural food supply grew arithmetically. This inability of the food supply to keep up with population growth led to cycles of feast and famine, as increases in human fertility led inevitably to crises of population mortality and decimation. These forces could be compounded by problems in legal institutions, such as the tragedy of the commons under which ill-defined property rights led to overgrazing and further worsening of the food supply.

Boserup's key contribution was to challenge the inevitability of Malthusian cycles. Population pressures on arable land, she noted, would lead to the use of labor-intensive technologies that would improve agricultural productivity. Increased productivity would in turn fuel improvements in infrastructure to permit improvements in harvest and distribution. As she describes:

If local population increase provides the incentive for an expansion of the productive capacity of agriculture, labor-intensive investment can remove the constraint on output by the limited supply of arable land and capital. Therefore, in periods of rapid population growth, investment in agriculture by direct labor inputs is at higher levels than in periods of low or negligible rates of growth of local population. This is true not only of investment in traditional food production, but also in production of special export crops (Boserup, 1975).

This dynamic is the basis for induced technological change: “intensification is an efficient response to the rising rental value of land relative to wages (Roumasset and Smith, 1981).” Induced technological change, however, is costly. Low levels of wealth and limited access to capital would create risk aversion leading to economically rational resistance supporting “technical inertia” (Wood, 1998). Another limit on induced technological change is existing economic infrastructure. In addition to risk aversion, Boserup notes, that individuals “may have insufficient incentive to produce a surplus beyond subsistence needs because the lack of infrastructure results in high costs of transport and distribution both for locally produce agricultural products and for products imported in the area from outside (see Boserup, 1975, p. 260).”

Economic development in Europe offers support for Boserup's theory. Her analysis contradicts the assumption that in pre-industrial Europe, technological change was “random and too rare to have had much importance for population trends, until the great breakthrough of modern technology at the end of the eighteenth century” (Boserup, 1987). Population density was positively related to market access and the level of transport technology (Boserup, 1987, p. 695). Population size and density made concentration in urban centers possible, increasing the size of the intellectual elite (Boserup, 1987, p. 695). Such concentration also facilitated the creation of guild systems, family organization, and systems of marriage, sparking increases in savings and the accumulation of wealth (Boserup, 1987, p. 696–697). Boserup acknowledges that a triad of crises, epidemics, war, and famine, shaped the trajectory of population growth and density. But she questions whether these crises created “subsistence crises” as land resources served as a bottleneck to population pressure. According to Boserup, labor, not land, was the scarce resource as “rural labor supply could cultivate sufficient land with sufficient intensity to produce in normal harvest years (Boserup, 1987, p. 696–697).”

Several scholars have generalized Dr. Boserup's theory of induced technological change beyond agricultural economic development to integrate politics, ecology, economics, and technology studies. Some identified the endogeneity of “techno-managerial strategies of agriculture” in response to environmental and demographic changes which induced innovation and investment in technology (Turner and Fischer-Kowalski, 2010). While some have criticized Boserup's theory for ignoring the role of social institutions, Boserup's response was that social institutions, like technology, are endogenous to external factors like the environment, reflecting choices on how to organize society (Turner and Fischer-Kowalski, 2010). This strand of induced innovation theory supports ideas of social innovation in the design of institutions (Baglioni and Sinclair, 2018). Induced innovation, both technological and social, follow certain identifiable steps that can serve as a framework for policy reform and social change (Newig et al., 2019).

Induced innovation proffers a mechanism for generating a virtuous circle of abundance. Simon's triumph over Ehrlich is a popular cultural illustration of how technological change spurred by scarcity permits escape from scarcity's constraint. These are “fables of abundance,” to borrow a phrase from historian Jackson Lears, who identifies in early twentieth century advertising fantasies of industrial production, mechanization, and expanding civilization. Although writing about manufacturing, Lear's examples of fables of abundance have parallels in contemporary tales of the wonders of digitization's bounty, which I elaborate upon in the next section. Abundance and its supporting fables, however, needs, in the words of Professor Barbara Fried, to “face up to scarcity” (Lears, 1994; Fried, 2020). Theories of abundance fall into what Professor Fried calls non-consequentialist thinking, which reject utilitarianism's emphasis on aggregating individual interests in making policy choices. Like other non-consequentialist theories Fried identifies (Rawls, Nozick, Scanlon), theorists of abundance assume deontic claims that ignore trade-offs across individuals. Professor Fried's admonition aimed at non-consequentialists, applies as well to the cornucopians:

Virtually, all collective choices we make require us to trade-off one person's interests against another's….The essentially optimistic premise on which non-aggregation rests—that tragic choices between the fundamental interests of different individuals are the exception and not the rule—cannot tell us what to do about it (Fried, 2020, p. 3).

Abundance must also face up to scarcity.

Four dimensions define scarcity's showdown with abundance. The first are distributional concerns masked by cornucopianism. Next is scarcity as to ends in distributing the fruits of abundance. Third are the issues of management and sustainability needed to avoid wasting away abundance. Finally, there are the increased wants and needs induced by abundance.

Distributional concerns lead to the questions: abundance for whom and of what? Cornucopians seemingly view abundance in abstract social terms, as the creation of surplus that benefits individuals in the aggregate. This nod to aggregation is apparent in the Simon-Ehrlich debate and its focus on the price change on selected resources. A price drop, Simon deduces, is a sign of abundance leading to social benefit. But the use of these measures ignores the question of who benefits from abundance and how. Does an unlimited supply of consumer goods (cars, appliances, fashion) inure to everyone's benefit? The digital divide demonstrates inequities in an age of abundance, and, as I described below, an abundance of information does not mean equality of access or a shared ability to transform information into knowledge. Thomas Piketty documents movements toward more equal distribution of incomes and wealth across many countries. This “great redistribution” from 1914 to 1980, as he describes, is attributable to expansions in social welfare programs, progressive taxation, and the liquidation of assets and relief of public debate arising from decolonization (Piketty, 2022, p. 121). But inequities still exist, he notes, across nations and within nation states across the lines of class, race, and gender (Piketty, 2022, p. 45–47). Abundance has its limitations against standards of equality and fairness. How do we address trade-offs between abundance and distributional concerns?

Abundance leads to questions of distribution. As individuals witness abundance's bounty enjoyed by neighbors, envy induces the quest for a share of abundance's fruits. This quest leads to an important twist on Robbins' defining scarcity in terms of means. Abundance leads to questions of ends and how to spend the surplus a society enjoys. Should surplus be invested back to further induce innovation or should the surplus be used to sports arenas, theaters, schools, hospitals, or other list of needs and wants? Robbins would have classified these questions as noneconomic, perhaps the subject of politics or social mores. Within contemporary economics, social choice theory and public choice theory turn to questions of institutional design to allow social choices among these conflicting ends. Whatever disciplinary methodological is applicable to the problem, abundance must face up to the existence of scarcity in ends.

Scarcity threatens abundance in terms of management to sustain abundance's bounty. Political battles over the choice of ends may lead to a waste of abundance as interest groups may expend resources to gain a larger share of surplus than competitors. Rent-seeking, in various forms, drives the success of conflict ends. Social institutions can attempt to manage the uses of abundance and ensure the distribution of its benefits. Professor Elinor Ostrom's scholarship on the commons illustrates how social choices on institutional design arise to choose among conflicting ends, even in a world of abundance. Cornucopians need to face up to scarcity on how to manage abundance to reach socially chosen ends and to sustain abundance without wasting its bounty.

Finally, society needs to manage abundance to choose among scarce ends because need and wants increase in the face of abundance. Returning to Lears' fables of abundance, we can witness advertising creating new wants as supply generates demand. Scarcity exists not only in a world of deprivation but also in a world of plenty to satisfy the quest for more consumer goods, investment opportunities, and even newer things. Professor Whybrow's research, discussed in detail below, documents this perhaps less than virtuous circle for the pursuit of wants.

Against this background on debates over scarcity and abundance, we turn now to the example of time as an illustration of how scarcity and abundance serve as analytical and rhetorical tools for the regulation of information.

## Making time scarce, making time abundant

Scarcity and abundance are in tension within intellectual property law. This tension stems from that between limitation and freedom in law more broadly. However, within intellectual property law, particularly copyright law, we can see this tension as flowing from our attitudes toward time^3^.

Time can have many meanings relevant for my discussion here. A physical notion of time is relevant for understanding information processing and methods for collecting information. Biological notion of time informs how we live from mundane processes of sleep and eating to more long-term changes such as aging. Sociological time shapes our relations to others: anniversaries, milestones for children, daily needs. Engineering notions of time define clocks, whether mechanical, electronic, atomic, and astronomical. All of these notions of time are relevant for my points here although as the argument unfolds engineering measures of time might be the most salient.

Whatever notion of time we are using, it should be distinguished from labor, a concept more familiar for intellectual property and information. Locke's theory of property rests on appropriation through labor. But as may be familiar, a labor theory of property and, within economics, of value is inadequate for understanding questions of distribution attendant to property and markets. Within contemporary economic and sociological theories, labor is a question of how individuals allocate time for different activities. See, e.g., Becker (1965), Emens (2019).

Finally, my approach here is different from that of the Austrian School of Economics, whose followers start with theories of time as relevant to uncertainty and entrepreneurship. See Schulak and Unterkofler (2011). I discuss entrepreneurship and the Austrian School, with critical comments, in Shubha Ghosh, Advanced Introduction to Law and Entrepreneurship (2021). Here, my emphasis is on how different forms of regulation control the scarcity and abundance of time as illustration of how control of time is a form of information policy.

Abundance within intellectual property law follows from public goods theory. Writings, applied ideas, and the resulting domain of culture and science are often characterized as public goods, ones whose benefits are shared among groups of people and not limited to one's individual use (Ford, 2021). Scarcity appears in the possibility of congestion through overuse of existing books and knowledge without the replenishment of original creations and new inventions (Landes and Posner, 2003). Congestion is a type of cultural degradation and ennui reflected in the decrease in demand for public domain works reflecting the diminution in value. By contrast, the positive externalities that flow from the public goods of culture and science can spur further invention and innovation. Abundance is in a virtuous.

Circle (Cohen, 2011; Lemley, 2015). Scarcity interplays with abundance in trademark law as well. Some scholars teach us that the trademark registration system is running out of words. Language itself has its limits as a basis for indicating source and distinguishing products (Beebe and Fromer, 2018). Contra The Beatles, words no longer flow out “like endless rain into a paper cup (Lennon and McCartney, 1968).” However, new signifiers stem the scarcity through the cornucopia of trade dress, design, smells, haptics, sounds, and kinetics (Lukose, 2015). Non-traditional trademarks are abundant, limited perhaps only by the ability of administrative offices to keep up (Croze, 2018).

Scarcity and abundance have their analogs in exclusive rights and access. Exclusivity is about limitations, metering out uses based on the calculus of the rights owner. This calculus is an economic one not limited to material gain through royalties and transfers but also a moral one, reflecting distrust for certain uses as interfering with the moral rights of the owners. Access, by contrast, is about abundance. Future inventors can make improvements on existing inventions or they might make “one horse shays” obsolete. Musicians can transform compositions across genres. Parodists and satirists generate their commentary. Books are made into movies; movies into books. Access enables abundance through the virtuous circle of transformation. Within copyright law, fair use mediates scarcity and abundance as a justified limitation on exclusivity to facilitate new creative actors to participate within their artistic communities.

Monopoly and competition also have their roots in scarcity and abundance. As Robbins noted, copyright creates artificial scarcity that allows owners to allocate scarce uses relative to demand. This artificial scarcity works a monopoly in a legal sense as a limitation on competitive entry. Competition leads to abundance, for the utopians an unlimited one unconstrained by costs, scale, and demand. More realistically, competition enables an abundance relative to the scale of production, costs of making and distributing physical works, and the demand for them. In a digital environment, costs may be substantially lowered and economies of scale for distribution will be increased, but demand would still be a factor on how much abundance that can be enjoyed. Monopoly and competition are the institutional dimensions of scarcity and abundance, defining the shape and dynamics of a market in which transactions for sales, licenses, and other agreements operate. But within these institutions play out the psychology of scarcity and abundance, which inform the behaviors driving creation, invention, marketing, “trafficking and trucking” (to use a quaint vernacular). This psychology reveals the dynamics of scarcity and abundance and how they are regulated.

### Psychology of scarcity, abundance, and self-control

Journalist Michael Lewis in his comparative study of the economic downturn of the 2000's identifies the psychology of scarcity and abundance (Lewis, 2011). Drawing on the work of UCLA neuroscientist Whybrow (2005), Lewis starts from the proposition that “the human brain evolved over thousands of years in an environment defined by scarcity. It was not designed at least originally for an environment of extreme abundance” (Lewis, 2011, p. 204) Quoting Whybrow:

We are set up to acquire as much as we can of things we perceive as scarce, particularly sex, safety, and food….When faced with abundance, the brain's ancient reward pathways are different to suppress. In that moment the value of eating the chocolate cake exceeds the value of the diet. We cannot think down the road when we are faced with the chocolate cake (Lewis, 2011, p. 204).

Self-control is limited by the need to survive scarcity, whether real or feared. As explanation for the bust of the 2000's (and perhaps previous cycles of economic boom and bust), Lewis observes,

The richest society the world has ever seen has grown rich by devising better and better ways to give people what they want. … The succession of financial bubbles, and the amassing of personal and public debt, Whybrow views as simply an expression of the lizard-brained way of life…..The boom in trading activity in individual stock portfolios; the spread of legalized gambling; the rise of drug and alcohol addiction; it is all of a piece. Everywhere you turn you see Americans sacrifice their long-term interests for a short-term reward (Lewis, 2011, p. 205).

This inability to self-regulate, Whybrow predicts, leads to either excessive glut and self-destruction, even death, or a moment when the bottom falls out forcing a turn to external forms of regulation. “If we refuse to regulate ourselves, the only regulators are our environment, and the way that environment deprives us” Lewis quotes Whybrow (Lewis, 2011, p. 206). Lewis concludes: “For meaningful change to occur, in other words, we need the environment to administer the necessary level of pain” (Lewis, 2011, p. 204) Written around 2010, there is a foreboding quality to Lewis's analysis, suggesting to me why the move to forms of authoritarianism that some scholars have noted in modern world politics as an external substitute for the lack of internal self-regulation (Rhodes, 2021). The prognostication also is reminiscent of the doomsaying of Paul Ehrlich. Whybrow's insights not only enlighten the psychology of scarcity but also question whether abundance is a virtuous circle.

One dimension of contemporary abundance is what is popularly referred to as the information glut. If the lack of information, or ignorance, is a form of scarcity, then the information glut arises from a desire to hoard information, to stave off ignorance with perhaps the illusion of knowledge and enlightenment. This accumulation of information for its own sake may come at the expense of being able to distinguish good information from bad information. Understanding the information economy in terms of the interplay of scarcity and abundance provides the bridge between the discussion of Robbins and Boserup in Sections Two and Three of this Article with the discussion of information, communications technology, and copyright in the rest of this Article. One final piece is identifying how the psychology of scarcity and abundance connects to the constraints of time.

### Self-control and time

Behavioral economist Sendhil Mullainathan and psychologist Eldar Shafir collaborated to show how the psychology of scarcity perpetuates income inequality and derive policy recommendations to combat poverty^4^. Although their focus is on the poverty of income, their analysis has application to the poverty of information. This connection is based on what could be described through the cliché that “time is money.” In their book on scarcity (Mullainathan and Shafir, 2013), the authors relate one of their battles with battling deadlines. Despite being aware of the many obligations that Sendhil had taken on, obligations familiar to many of the readers of this Article, Sendhil nonetheless found it difficult to say no to other requests, whether committee meetings or contributions to a book. Furthermore, as the obligations accumulated, Sendhil used his precious time to complain about the lack of time to meet his deadlines. The stress of time was exacerbated by recognizing the lack of it. The authors draw a parallel between the lack of time and the lack of money:

Missed deadlines are a lot like overdue bills. Double-booked meetings (committing time you do not have) are a lot like bounced checks (spending money you do not have). The busier you are, the greater the need to say no. The more indebted you are, the greater the need to not buy. Plans to escape sound reasonable but prove hard to implement. They require constant vigilance—about what to buy or what to agree to do. When vigilance flags—the slightest temptation in time or in money—you sink deeper (Mullainathan and Shafir, 2013, p. 3).

Even though the woes of a harried academic seems on the surface remote from an indebted low income homeowner, they both are facing the problem of scarcity, which for their purposes the authors define as “having less than you feel you need.”

This succinct and clear definition relates to Robbins' definition as they both recognize scarcity as a problem of management. For Robbins, the problem is one of managing scarce means to satisfy certain ends. For Mullainathan and Shafir, scarcity points to a connection between time management and money management. What the contemporary authors note however, drawing on their work on psychology, is that scarcity as a management issue is connected to that of mental bandwidth. Just like a browser with multiple open windows:

Scarcity does something similar to our mental processor. By constantly loading the mind with other processes, it leaves less “mind” for the task at hand. Scarcity directly reduces bandwidth – not a person's inherent capacity, but how much of that capacity is currently available for use (Mullainathan and Shafir, 2013, p. 39).

From this analogy, they identify a possible pathway for policy reform to address poverty. Instead of viewing poverty as a failure of character or poor time management as akrasia, the authors conclude:

The scarcity mindset, in contrast, is a contextual outcome, more open to remedies. Rather than a personal trait, it is the outcome of environmental conditions brought on by scarcity itself, conditions that can often be managed. The more we understand the dynamics of how scarcity works upon the human mind, the more likely we can find ways to avoid or at least alleviate the scarcity trap (Mullainathan and Shafir, 2013, p. 123).

Confronting the problems of scarcity is a matter of environmental design. Reforms should target the limitations that scarcity places on cognitive function and management. As Mullainathan and Ershad focus on poverty and income inequality, their reform proposals focus on changes to the welfare system to permit better transition to work and time management in job training programs. Beyond the application to welfare reform, their insights on scarcity as a problem of the environment is useful in understanding reforms to address information poverty.

Information poverty describes a situation arising from the information glut of the digital economy. As more digitization has led to an abundance of websites, podcasts, visual content, electronic books, and streaming music, all easily available for a subscription, through shared services, like YouTube, or *via* surreptitious means, the typical consumer finds themselves unable to process and distinguish among all the options. What results are not the congestion costs identified by Landes and Posner but an overwhelming fear of not being able to keep up as the information overload blurs the lines between reality and fantasy, quality and fluff, true and false. Information becomes a sort of junk food, plenty of options but with little nutrition. As economist Daniel Hammermesh described: “Our ability to purchase goods and services has risen much more rapidly than the amount of time available for us to enjoy them (Hamermesh, 2018, cited in Krueger, 2019).” Goods and services grow exponentially while time increases arithmetically in a rat race of increased productivity and increased labor at the expense of leisure. In the information age, it can take a lot of work to be a channel surfer, sorting through the program guides, figuring out the remote, keeping track of all the subscriptions and saved programs.

Economist Alan Krueger traces the poverty of plenty to John Maynard Keynes, who imagined the future of his grandchildren (meaning us):

Thus, for the first time since his creation man will be faced with his real, his permanent problem—how to use his freedom from pressing economic cares, how to occupy the leisure which science and compound interest will have won for him, to live wisely and agreeably and well (Keynes, 1930, p. 267).

In an age of abundance, scarcity is measured by the extent of our want and ambitions, rather than our subsistence needs. Keynes foresaw that “it will be those people, who can keep alive, and cultivate into a fuller perfection, the art of life itself and do not sell themselves for the means of life, who will be able to enjoy the abundance when it comes (Keynes, 1930, p. 268).” But as the current economic psychology literature teaches, abundance can lead to a loss of self-regulation and scarcity can create cognitive failure as time becomes the taunting constraint. Robbins posited constraint as a problem of management, of choosing among scarce means to reach our desired goals. Mullainathan and Ershad update this problem of management in terms of choice architecture. Keynes would view this management of time and the architecture of choice through government intervention transforming the freedom from need into the freedom to enjoy abundance.

### Regulating and deregulating time

How do we understand the management of time within law and policy? At a basic level, management of time stems from a sense of mortality and the accompanying survival instinct. A natural response perhaps is to escape time itself through expanding it or chasing immortality. Increases in life expectancy through medical technology and lifestyle management has made time a less binding constraint, but only to a point as the bucket list simply grows longer. Legal mechanisms exist for simulating immortality. The corporate form allows for perpetual existence if not of the human body but of its manifestation in artificial form^5^. Various forms of dead hand control through bequests, trusts, conditional gifts, and philanthropy also simulate immortality through the dream of perpetual management. Limitations on dead hand control, however, allow for new generations to throw off the yoke of tradition (Radin, 2011). Management of time is, as with any resource, subject to competition among conflicting actors.

Time management has a well-known legal foundation, one that explains much of the current information glut and information poverty. In its Sony v. Universal Studios decision, the Supreme Court in a 5-4 judgment recognized time shifting as fair, and substantially non-infringing, use under copyright law (Sony Corp. of America v. Universal City Studios, 1984). Although the legal doctrine has echoes of science fiction, the Court was not acknowledging the existence of the Tardis. Instead, the machine the five justices were saving from the damnation of secondary liability was the videocassette recorder (in the more efficient, but soon obsolete form of the Betamax). What the VCR allowed was escape from the limitations of broadcast time of television programming. Before the VCR, viewers would have to be in sight of a television set to catch a program at a particular time, whether in a house, in a hotel, or in front of a department store window. Such a constraint affected not only viewers but also merchants. For example, famously shopping and restaurant dining dropped precipitously in the mid-1950's on Mondays at nine o'clock in the evening when “I Love Lucy” was broadcast. By permitting taping for later viewing, the recorder opened the market, not just for broadcast television viewing but for other activities. Nearly forty years after the decision, the average person can watch a recorded program on a tablet while trolling websites for the best bargain and ordering dinner on one's laptop as the latest multipart drama plays out on the big screen Sony television. Keynes might be proud of his foresight.

Network broadcasting in the 1950's made time a scarce resource. Time shifting^6^ made time abundant. Scarcity and abundance here are relative to the needs of television viewing, to be sure, but the constraint and its release had implications beyond the sanctity of one's couch, armchair, or bed (Samuelson, 2007). The Court's ruling in Sony extended other liberating aspects of technology. Time shifting as fair use saved the Diamond Rio, the not-so-distant ancestor of the iPod which begat the iPhone and iPad. As broadcast television markets expanded so did the entrance of new television stations, entry made possible through reforms of telecommunications law and advancements in digital and satellite technologies. Time shifting allowed for more options for a typical evening's entertainment beyond scheduled programming, and the accompanying technological advances allowed for the entry of new forms of entertainment beyond the dictates of the dominant television and radio networks. Today we witness the cornucopia of social media and streaming services. In this time of abundance, time once again becomes a scarce resource and with that scarcity comes the question of management and cognitive constraint. This current dialectic of scarcity and abundance calls for consideration of the legal, economic, and social architecture of management, the focus of the next and penultimate section of this Article.

## Regulating the information glut through time architecture

By recognizing time shifting in its Sony decision, the Court recognized the possibility of individual choice in viewing content made possible by the videotape recorder. Audiences were not limited by the constraints imposed by broadcast television. Accompanied by technological changes^7^ in cable, satellite, and digital transmissions, the possibilities for time shifting opened a vast content market, a cornucopia of information, entertainment, and self-expression.

But this abundance is illusory. Even with the possibilities of multitasking that allow for more intensive uses of viewing time (multiple windows on the browser, multiple devices on simultaneously), the expanded possibilities of time give way to the limits of attention. As attention becomes the new constraint, the ability to assess information, to distinguish between factual news and fictional entertainment, and to think critically about what one experiences confronts the limits of informational entropy. Removing one source of scarcity rebounds into the creation of other constraints against periods of abundance.

Against this illusion of abundance, calls for various types of regulation point to a need for new architecture for managing attention against misinformation, hurtful speech, propaganda, and other corruptions in an unregulated content market. This section makes the argument that what these several proposed reforms share is a mechanism for making time scarce, placing limits on its abundance in order to permit focused attention. Although the strictures of broadcast time cast off through time shifting were too rigid, they did impose a seemingly attractive structure, limiting choice but preventing overload. Modern regulatory approaches, I argue, seek to channel the freedom afforded by time shifting through targeted scarcity that controls the unfettered sprawl of abundance. After a consideration of self-regulation, I identify four types of time regulation: (1) delaying posts, (2) compartmentalization, (3) velocity and acceleration (with nudging as one example), and (4) reviving the spirit of the fairness doctrine. I conclude with the point that while these proposals do help to identify salient features of time architecture, focusing solely on time architecture should not distract from other policy concerns, such as directly confronting the harms created from pollution of content.

### Self-regulation and its limits, with the example of Wordle

An immediate inclination to controlling information glut is self-regulation, which entails placing the burden on content users (whether on social media, various internet platforms, numerous media providers such as cable or streaming) to manage their consumption of content. To revert back to Whybrow's analogy to chocolate cake, self-regulation is a self-imposed regimen of diet, exercise, and information abstinence. This regimen would include strategies such as scheduled viewing, limits on devices, content blockers, or discrimination choices of platform selection. It would also require self-education and vigilance to become informed on how to read posts, how to gauge the veracity of information, and how to glean content creators. We can describe self-regulation as effective forms of time management, knowing when to just turn the noise off and find shelter in modulated silence.

Within the language of time management, self-regulation has an analog within retirement planning in the shift from defined benefit to defined contribution accounts^8^. While defined benefit plans are employer managed pensions, managed centrally as a promise to provide a certain annuity payout, defined contribution plans are employee managed, building on contributions from salary, sometimes matched by the employer. In contrast to defined benefit plans, defined contribution plans require retirement savers to be proactive in making financial decisions from how much to save to when to require. Defined contribution plans, however, raise questions on the ability of future retirees to self-manage their retirement plans. The fields of behavioral economics and behavioral psychology grew through identifying the limits of rationality that can lead to failure to save adequately for retirement. These failures led to policy reforms of the architecture of savings through such reforms as opt-outs, nudges, or the design of retirement securities (Jolls et al., 1998; Benartzi and Thaler, 2007).

By analogy self-regulation of time would also require careful consideration of time architecture. For self-regulation to work effectively, content users need to have the knowledge to judge content and the time to remain knowledgeable and assess the information onslaught. It takes time to manage time; it takes information to understand information. How time is structured can affect how effectively it is managed. A rigid structure, the paradigm of working nine-to-five under a strict regimen, is one possible architecture, but a possibility that would take away choice and freedom. But there are other possibilities.

Take the example of Wordle. An Internet and social media phenomenon, launched in late 2021, Wordle illustrates time architecture that focuses attention and manages time in game playing. A daily challenge posted every day after midnight New York time, the game provides six chances to guess a five letter English word. (There are versions in other languages). The only time limit is the launch of the next game (although in theory one could take forever to solve a single game by adjusting one's browser) and so the solution is self-paced. The main reward is finding the solution in the fewest number of tries, with two or three being the gold standard and one guess being the sign of good luck. Wordle's time architecture allows for self-regulated and focused attention, promoting concentration and mental exercise. Its success had been imitated in forms with similar architecture, such as Heardle (for identifying musical segments), Globle (for identifying geographic boundaries), and Semantle (for identifying words related semantically).

Wordle's appeal is an example of what social psychologist Mihaly Csikszentimihalyi calls flow, a process of total involvement with life that exhibits the joy and creativity of human life. As he points out, “jobs are easier to enjoy free time because like flow activities, they have built-in goals, feedback, rules, and challenges, all of which encourage one to become involved in one's work” (Csikszentimihalyi, 1990). Wordle's design provides the requisite feedback, rules, and challenges to bring the joy of flow to free time. Architecture regulates free time, providing a light-handed regimen that channels one's play into nuggets of engagement.

While Wordle illustrates how architecture supports self-regulation, a recent episode also shows why focusing solely on time architecture is inadequate for regulating the problems of the information glut. In May, 2022, The New York Times, which now owns and manages Wordle altered the programmed word of the day to avoid the perception of its using the game platform to promote an editorial message. The word at issue was “fetus,” a term of medicine and biology made controversial by the abortion battles and the pending reversal of the precedent, Roe v. Wade. As replacement for this “f-word,” The Times substituted “shine,” a seemingly neutral and joyful alternative. This on the surface innocuous episode demonstrates that focusing on time architecture alone can cloud questions of algorithm regulation and content moderation. Altering a word not only confused Wordle players that day but also raises the question of whether time architecture in promoting scarcity is responsive to problems of information abundance, the ultimate lesson of this Section.

### Reform proposals and the architecture of time

Broadcast television before the private home use of the VCR structured time for home viewing of television content. This regimen standardized time much in the same way other industries, such as the railroad, shipping, or telegraphy, standardized time to facilitate transactions. Standardizing time has been a means of regulation for the military, for the workplace, and for the administration of colonies. As demonstrated above, the dissemination of the VCR, with the aid of the Supreme Court's 1984 ruling, liberalized time in the broadcast space, paving the path for various technologies that allowed for more time-flexible communications and information sharing.

Liberalization of time combined with the new communication and information technologies has led to an abundance of information which has created new sources of bottlenecks on time. Reform efforts to address the information glut stem from the limits of self-regulation. In assessing these reform efforts, I make the case that reform proposals can be understood as new ways of regulating time without reverting to the rigid standardization that existed in the pre-VCR period. New time architectures are at the heart of these proposals. Assessing these implicit time architectures will enlighten some of the limits of the proposals.

#### Delaying and limiting posts

One way to regulate information overload is to delay the timing of posts and limiting the number of times a user can post content. Delaying posts allows for more deliberation in commenting on content and slows down reactive and emotional responses. Limiting the number of posts also can induce efforts to improving the quality of posts. Delays and limits are examples of imposing scarcity on time by restricting the amount of usage. They are analogous to character restrictions on Twitter, another form of constructed scarcity. Each impose a regimen on users with the result of reducing the demands on the processing and accessing of information.

Delays and limits are illustrations of what some scholars may call frictions^9^ and raising transactions costs (Driesen and Shubha Ghosh, 2005; Fennell, 2009). When understood as frictions, these reform proposals may appear similar to the proposals on the velocity of information, discussed below. But time delays and limits are also closer to structured time of broadcast television, pre-time shifting. Users are limited as to when and how often they can engage with content. But delays and limits allow some degree of time shifting since users are still allowed to choose their own schedule for creating, viewing, and responding to content. Therefore, delays and limits impose scarcity on a world of time abundance. In the language of Mullainathan and Shafir, delays and limits impose some degree of time flexibility by allowing users to decide when to spend the restricted time they are granted. Users can enjoy a rationed form of abundance.

#### Compartmentalizing time

Time architecture also imposes a schedule on how time is used. Within the military, for example, there is a time for exercise, a time for eating, a time for grooming, and a time for sleeping. Such strictly compartmentalized time is reflected in the world of pre-time shifting broadcast in categories like “Prime Time,” “Children's Viewing,” or “Adult Programming.” Compartmentalizing time imposes scarcity on abundance like delays and limits, but in a more structured way. The contemporary proposal building on “attention accounts” is an illustration of compartmentalizing time.

Professor Cass Sunstein points to an attention deficit as potentially subverting the management of information through disclosure requirements and regulation of communications technologies:

There are serious limitations on the amount of information to which people can attend at any point in time. The standard economic account would emphasize that attention is a scarce recourse and suggest that people make rational (even if fairly rapid) decisions about how to allocate it. Research in psychology, by contrast, suggests that people do not decide how to allocate attention; certain items capture attention while others disappear into the background, even if they are exceedingly important and even if it would be rational to focus on them (Sunstein, 2020).

Information management rests on “attention accounts,” some data are given more weight than others and some are ignored all together. Gathering and use of information is often instrumental, and people process what they know based on what they need to know to reach financial goals, a specific grade in a class, entry into a profession, determine how to vote, and other decisions that people must make. Information also may be obtained for purely aesthetic ends. Examples of this might include gossip, historical or geographic trivia, and engagement of the imagination and fantasy (think of the thrill over guessing or understanding the ending of The Sopranos). These many dimensional benefits of information should shape the economic, social, and legal architecture of information management. Disclosure requirements should be clear and easy to understand. Regulation of social media should keep in mind the various uses of information platforms, as a source of news and a source of distraction. Welfare analysis of regulation, Sunstein argues, needs to account for these complex benefits as well as the costs of what I have called information glut and information poverty.

#### Nudging and informational velocity

Time architecture can be dynamic in addition to the static design of delays, limits, and compartmentalization. The familiar nudging is an illustration of how choice design includes a dynamic push or pull toward a desired outcome (Thaler and Sunstein, 2021). We can think of a nudge as controlling the speed through which choices are made. Instead of requiring an instantaneous decision, regulation can push or pull gradually toward correct choices about such matters as retirement planning or selection of information content. Information appraisal can be made deliberate, requiring users to review content slowly and with care. Time can be slowed down, but it can also be speeded up. The latte design may be relevant in order to avoid clearly erroneous content or to act quickly in response to emergencies or other information warnings.

Nudges and the speed of time are connected to what Professor Daniel Kahneman describes as “thinking fast” and “thinking slow” (Kahneman, 2013). These are hardwired cognitive functions that reflect different modes of responses to different situations. Thinking slow and fast are the source of identified cognitive failures, such as the endowment effect, availability bias, or recency. Time architecture seeks to regulate these cognitive functions in contexts involving the management of risk and the dilemma of uncertainty. Thinking fast and slow are relevant to the risks and uncertainty associated with information content. Time architecture can induce slow thinking or fast thinking through warning signs, such as color-coded labels for various forms of information. Ratings can also assist in slow and fast information processing through identification of adult or child-friendly content. Instruments for guiding information management can create nudges toward desirable content, promoting either slow or fast thinking and the regulation of the speed of content consumption.

The term velocity is more appropriate than speed in this analysis. A vectored value of speed, velocity has both magnitude and direction. For time architecture, direction needs to be considered as it points to the goals of the regulation. What is the end to which a nudge leads? The answer to the question entails a normative judgment that goes beyond the technical aspects of time architecture which has been the focus of discussion. The direction component of velocity connects time as an instrument for the regulation of information. As I conclude in this section, imposing scarcity on the abundance of time can distract from challenging questions of content moderation and speech. Nudging and velocity reveal some of the limitations of time architecture, a point that is developed in the following subsections on vanishing content and the scholarship on the fairness doctrine.

As velocity speeds up time, acceleration also arises in proposals regarding time architecture and information regulation. A brief discussion here of ephemeral content illustrates another dimension of time architecture. Some platforms present content with an expiration date; its content vanishes and is unretrievable after some amount of time. This design is the obverse of time delays and limits as it accelerates time requiring faster viewing of content and almost no time for response. Accelerating time prevents content from lingering and having a long-term effect on users, who either see the content or miss it. But ephemeral content is undesirable for many reasons^10^. Memories are lost. Cumulative understanding becomes impossible. The public domain vanishes with the removed content. While vanishing content may prevent persistent misinformation, it is a bad design of time architecture and illustrates an extreme form of constructed scarcity as a cure to information glut.

#### The fairness doctrine and the dimensions of scarcity and abundance

Time architecture imposes new types of scarcity on the abundance of time, one that in some forms tries to recreate the extreme regime of broadcast pre-time shifting. In this subsection, I connect time architecture back to the discussion of scarcity and abundance in the first part of this Article in order to show the problems of scarcity and abundance rhetoric. My conclusion is that time architecture built on scarcity distracts from challenging questions of content moderation and free speech.

My dialectic approach to scarcity and abundance as illustrated through time architecture casts light on media regulation and First Amendment. Scarcity based justifications for media regulation, whether looking at the limits of the spectrum or platforms, are too simplistic, ignoring the social construction and malleability of scarcity^11^. Those who support the traditional Fairness Doctrine or its updated versions, for example, will have to look at broader and deeper justifications than notions of scarcity. Similarly, those who appeal to abundance to counter antitrust scrutiny of media platforms (for example because of adequate potential competition or abundant consumer options) need to consider how scarcity continues behind the veneer of abundance. Scarcity and abundance are distractions from more subtle policy concerns, such as how to educate the public to critically assess content as well as how to maintain a robust and diverse market for content. What my analysis calls is for a richer institutional analysis of media regulation and the First Amendment, as we see, for example, in Martha Minow's new book, *Saving the News* (Minow, 2021).

For example, the Fairness Doctrine requiring equal time for alternative perspectives in the presentation of the news rested on the scarcity of broadcast frequencies that stemmed from the limitations from the radio spectrum. Since the government had to license these frequencies to avoid congestion, the power to license supported regulation to ensure equality of representation. Although the Supreme Court ruled the FCC had the authority to implement the Fairness Doctrine, the FCC eliminated the Doctrine in the 1987^12^. In part, this repeal was made possible by technological changes that undercut the scarcity rationale for the agency authority:

The rise first of cable and then of the internet altered the regulatory predicate of scarce speech opportunities and to some, reduced the need for a policy requiring balance within one outlet. Yet a deeper explanation for the end of the Fairness Doctrine lies in the erosion of public interest ideal in medial and in the country as a whole (Minow, 2021, p. 68).

Technology liberating radio and television broadcast from the scarcity of the spectrum. As a result, broadcast abundance provided opportunities for new entrants to reflect a range of perspectives. But as the market expand, new entrants were able to invest intensively in their individual market niche. There was no need to appeal to the public as a whole. With a differentiated product, a particular program need only appeal to a segment of the public to be profitable and have a prominent market position.

With abundance comes a deficit of time. Competition, whether in the actual marketplace or in social interactions, is over time both in its personal use and in its capture by those who seek it: advertisers, content producers, spreaders of news and rumor, reputation makers. Professor Minow identifies the conflicts between private attention grabbing and the public interest:

When it comes to digital platforms, as long as a combination of advertising and subscription determines the revenues, and as long as competition for those revenues leads to heightened rather than lessened efforts to gain user attention and user behavior, unethical behavior can easily follow (Minow, 2021, p. 119).

Fraud in the collection and use of data is one type of unethical behavior. Bias in content moderation as platforms cherry pick what posts to block or what to promote is another. Corruption within social platforms as users ignore the biases and accept the potential abuses because of pressures to conform would be the ultimate unethical behavior, undermining even the possibility for reform.

Antitrust is often touted as one reform measure to stem the time of unethical behavior. Scrutiny of advertising and subscription markets for anticompetitive conduct and unfair and deceptive business practices are necessary to combat fraud and consumer harm in these markets. But increased competition is a misguided response if competition for attention occurs through unethical practices. Fifty social platforms may not resolve the problem if they each act like the single platform does now. Heightened competition might lead to a race to the bottom in business practices.

Independent content moderation is needed to separate the moderation function from the content and revenue generation functions of social platforms. But the difficult question is designing the institutions for content moderation. Ratings agencies can serve as a watchdog as they do in the financial sector and in consumer protection. In theory, there is a potential market for ratings agencies to emerge to oversee social media platforms as to their accuracy and fairness. But the problem is to ensure that these agencies remain independent and not captured through the same forces of advertising and subscription revenues. Who governs the ratings agencies? Governmental standards, through certification and review, may reign in corrupting influences in the market for ratings.

As Professor Minow advocates, transparency in the architecture of social media platforms is necessary to regulate information management by users. Transparency extends to data collection and use as protections for information privacy as well as to the protection of consumers from confusing and misleading information generated from platform users. While information privacy can be policed through protections against unfair and deceptive business practices, protection against fellow users is fraught with difficulty. Professor Sunstein's points about the psychology of attention and the broader points about scarcity and abundance come into play. Users of platforms need to protect themselves from what in the real world is known as “stranger danger.” But protection from potential pickpockets and conmen is easier in a world of physical interactions than in the world of anonymous or pseudonymous interactions of social media. Self-help can only go so far. Social media architecture may need to police identify verification to prevent improper and illegal behavior as well as to punish it.

A more public minded approach to information policy needs to replace current decentralized and libertarian practices. For Professor Minow, this shift requires refashioning First Amendment as a limitation on government action to regulate speech as an affirmation of government policy to promote speech. Here, we return to the point with which we began this Article. Law limits freedom but can also affirm freedom. In the realm of speech, regulation of speech can make the market for speech more robust. Rules preventing fraud and deception can promote trust in the market. Governing content moderation, appropriate antitrust intervention, and rules on transparency are practical considerations to correcting the information poverty that stems from information glut. These proposals reach beyond scarcity and abundance.

#### The limits of reconfiguring time architecture

To summarize, time is a significant part of the architecture for information creating and sharing. Sometimes, time is made scarce by rationing when information is made available such as in the days of broadcast media before time shifting. With analog and digital technologies that permit various degrees of time shifting, time is made abundant in the sense that users have a choice of when and how to access content. In a world of time scarcity, time is a scarce means to distribute information. In a world of time abundance, we must confront the scarcity of conflicting and multiple ends, with attendant questions of distribution, that compound difficult choices of how time is to be used. Time is an illustration of the dynamic of scarcity and abundance that I set forth in the first part of this Article.

Our current age of time abundance, I have suggested, has led to the exponential growth of information through various forms of content: movies, podcasts, blog posts, social media uploads, ubiquitous photos and videos recording every thought, movement, and feeling. Current debates about how to regulate this information overload to prevent the dangers of fake news, harassment, unwanted content, and information theft entail to various degrees a regulation a time, with perhaps the world of time rationing through regimentation. These proposals, when cast in terms of reframing time architecture, I have argued in this section, are limiting. While the various proposals recognize the limits of self-regulation and individual choice in time management, they rely on a technical approach to rationing time to avoid difficult political choices about content and viewpoint. I am not recommending that we abandon these proposals. But we should approach them with clarity about how their implicit assumptions and their implementation.

Once we understand the problem of information overload in terms of time architecture, as it has transformed with developments in technology, we can better understand how we have arrived at our current media ecosystem. My analysis in this section has addressed the various approaches to redesigning time architecture as a technical matter of regulation. But my analysis also reveals the not fully understood connection between time and information. Information rationing and glut are related to the scarcity and abundance of time. Reconfiguring the architecture of time, however, can only partially address the challenges of information. I conclude this Article by pointing to research and regulatory questions after scarcity and after abundance.

## Beyond scarcity and abundance

This Article has made two overarching arguments: one about scarcity, abundance, and regulation generally and a second about time as an instrument of regulation subject to terms of scarcity and of abundance.

The first argument is that scarcity and abundance are rhetorical constructs that inform different regulatory institutions. Scarcity traditionally has mapped onto limits on freedom. Abundance, by contrast, props freedom's unlimited potential. Under the language of scarcity, limits promote outcomes, for example through rights to exclude, deprivation of a benefit, or imposition of a burden. Under the language of abundance, identified freedoms promote outcomes through rights of access or rights to use. Scarcity is distinct from absolute deprivation, and abundance, from unbounded and infinite possibility. Each are building blocks understood relative to the goals of institutional design. Furthermore, scarcity and abundance have an intertwined relationship, a dialectic of famine and plenty. Similarly, freedom and limitations coexist each supporting the other.

The second argument of this Article is that time as an instrument of regulation illustrates the uses of scarcity and abundance. Time can be regimented to regulate activities such as work, travel, diet, reproductive rights, social relations, and interaction with media. Time can also be liberating, seemingly abundant using perpetuities, technologies for fast forwarding, rewinding, or shifting content, and increases in the velocity of access and movement. Information retrieval, processing, and sharing are connected to time. It is no surprise that reform proposals for the problems confronting the information economy rest up regulation of time. This Article has demonstrated what these reform proposals share is an attempt to make time scarce, to return to perhaps an idealized era of regimented broadcast within an era of multivalent technological means for information creation and dissemination. But imposing scarcity on abundance ignores the deeper challenges of information glut and distortion: how to manage and assess content. This challenge also intersects with our understanding of time but cannot fully be addressed through concepts of scarcity and abundance alone.

In short, time as an instrument of regulation can have play in our design of regulatory institutions. But seeking to regulate through constructed scarcity or constructed abundance has its limits. As we continue to discuss information and its discontents, we need to see beyond the isolated categories of scarcity and abundance as we transform what we have into what we need. What lies beyond scarcity and abundance is a careful analysis of how our institutions are constituted to give play to the needs of freedom, social communication, political engagement, and thriving. Time, scarcity, and abundance are a small part of this broader endeavor.

## Data availability statement

The original contributions presented in the study are included in the article/supplementary material, further inquiries can be directed to the corresponding author.

## Author contributions

The author confirms being the sole contributor of this work and has approved it for publication.

## Conflict of interest

The author declares that the research was conducted in the absence of any commercial or financial relationships that could be construed as a potential conflict of interest.

## Publisher's note

All claims expressed in this article are solely those of the authors and do not necessarily represent those of their affiliated organizations, or those of the publisher, the editors and the reviewers. Any product that may be evaluated in this article, or claim that may be made by its manufacturer, is not guaranteed or endorsed by the publisher.

## Footnotes

^1^And perhaps a frivolous one now. As I understand it, despite some dissenters, the accepted view is that Shakespeare is the author of the plays in questions (although there may be issues of co-authorship). See, e.g., McCrum (2020).

^2^See, e.g., Rahmatian (2011) and Bracha (2016); For further background on the economics of copyright, especially the connection between public goods and monopoly, see Hadfield (1988). For an analysis of economic methodology and intellectual property, especially the importance of consequentialist thinking to intellectual property policy, see Ghosh (2021b).

^3^Time can have many meanings relevant for my discussion here. A physical notion of time is relevant for understanding information processing and methods for collecting information. Biological notion of time informs how we live from mundane processes of sleep and eating to more long-term changes such as aging. Sociological time shapes our relations to others: anniversaries, milestones for children, daily needs. Engineering notions of time define clocks, whether mechanical, electronic, atomic, astronomical. All of these notions of time are relevant for my points here although as the argument unfolds engineering measures of time might be the most salient. Whatever notion of time we are using, it should be distinguished from labor, a concept more familiar for intellectual property and information. Locke's theory of property rests on appropriation through labor. But as may be familiar, a labor theory of property and, within economics, of value is inadequate for understanding questions of distribution attendant to property and markets. Within contemporary economic and sociological theories, labor is a question of how individuals allocate time for different activities. See, e.g., Becker (1965), Emens (2019). Finally, my approach here is different from that of the Austrian School of Economics, whose followers start with theories of time as relevant to uncertainty and entrepreneurship. See Schulak and Unterkofler (2011, p. 33). I discuss entrepreneurship and the Austrian School, with critical comments, in Ghosh (2021a). Here, my emphasis is on how different forms of regulation control the scarcity and abundance of time as illustration of how control of time is a form of information policy.

^4^See excerpt from Scarcity available at: https://behavioralscientist.org/scarcity-excerpt-mullainathan-shafir/ (viewed on December 29, 2021).

^5^See Kantorowicz (2016). For the dominance of artificial persons, see Galanter (2006).

^6^See Volk (2008), available online at https://ininet.org/the-betamax-case-and-the-history-of-time-shifting-copyright-le.html (tracing time shifting back to broadcast industry usage in the 1950's). This control over time has its roots in the nineteenth Century with the expansion of the railroads and the need to standardize time nationally. “In 1883,…these ‘distinct private universes of time' [namely the time and the household] vanished when the railroads by joint decision, placed the country—without act of Congress, President, or the courts—under four standard time zones.” Stiles (2009), citing Trachtenberg (1982).

^7^In the background of course is the abundance in computing speed made identified as Moore's Law. See Rotman (2020).

^8^See Kotlikoff (2022) (short discussion of defined benefit and defined contribution plans).

^9^See, e.g., Brett Frischmann and Susan Benesch, friction-by-design regulation as twenty-first century tpm (unpublished manuscript made available by author).

^10^For a generalization of ephemeral content to disappearing content, see Lemley (2021).

^11^See Red Lion Broadcasting Co. v. Federal Communications Commission (1969) (“Because of the scarcity of radio frequencies, the Government is permitted to put restraints on licensees in favor of others whose views should be expressed on this unique medium. But the people as a whole retain their interest in free speech by radio and their collective right to have the medium function consistently with the ends and purposes of the First Amendment. It is the right of the viewers and listeners, not the right of the broadcasters, which is paramount”). But see Turner Broadcasting System (1994) (scarcity rationale for agency regulation of broadcast does not apply to cable television); Satellite Broadcasting Communications Association of America v. Federal Communications Commission (2001) (applying the reasoning of Turner Broadcast to agency regulation of satellite television).

^12^For discussion of the FCC's decision to not enforce Fairness Doctrine, and accompanying issues of FCC's rules on personal attacks and political editorials, see Radio-Television News Directors Association v. Federal Communications Commission 184 F.3d 872 (1999).
